# Chemical Source Localization Fusing Concentration Information in the Presence of Chemical Background Noise †

**DOI:** 10.3390/s17040904

**Published:** 2017-04-20

**Authors:** Víctor Pomareda, Rudys Magrans, Juan M. Jiménez-Soto, Dani Martínez, Marcel Tresánchez, Javier Burgués, Jordi Palacín, Santiago Marco

**Affiliations:** 1 Signal and Information Processing for Sensing Systems, Institute for Bioengineering of Catalonia, Baldiri Reixac 4-8, Barcelona 08028, Spain; vpomareda@ibecbarcelona.eu (V.P.); rmagrans@ibecbarcelona.eu (R.M.); jmjimenez@ibecbarcelona.eu (J.M.J.-S.); jburgues@ibecbarcelona.eu (J.B.); 2 Department of Engineering: Electronics, Universitat de Barcelona, Martí i Franqués 1, Barcelona 08028, Spain; 3 Department of Computer Science and Industrial Engineering, Universitat de Lleida, Jaume II 69, Lleida 25001, Spain; dmartinez@diei.udl.cat (D.M.); mtresanchez@diei.udl.cat (M.T.); palacin@diei.udl.cat (J.P.)

**Keywords:** machine olfaction, odor robots, chemical sensors, Bayesian inference

## Abstract

We present the estimation of a likelihood map for the location of the source of a chemical plume dispersed under atmospheric turbulence under uniform wind conditions. The main contribution of this work is to extend previous proposals based on Bayesian inference with binary detections to the use of concentration information while at the same time being robust against the presence of background chemical noise. For that, the algorithm builds a background model with robust statistics measurements to assess the posterior probability that a given chemical concentration reading comes from the background or from a source emitting at a distance with a specific release rate. In addition, our algorithm allows multiple mobile gas sensors to be used. Ten realistic simulations and ten real data experiments are used for evaluation purposes. For the simulations, we have supposed that sensors are mounted on cars which do not have among its main tasks navigating toward the source. To collect the real dataset, a special arena with induced wind is built, and an autonomous vehicle equipped with several sensors, including a photo ionization detector (PID) for sensing chemical concentration, is used. Simulation results show that our algorithm, provides a better estimation of the source location even for a low background level that benefits the performance of binary version. The improvement is clear for the synthetic data while for real data the estimation is only slightly better, probably because our exploration arena is not able to provide uniform wind conditions. Finally, an estimation of the computational cost of the algorithmic proposal is presented.

## 1. Introduction

Localization of chemical sources in urban scenarios (large cities) is a major challenge for intelligence and police authorities. In the clandestine production phase of illicit substances like explosives, but also drugs, significant levels of precursors are spread in the atmosphere. Such suspicious compounds could be reported by a system of mobile sensors and could be located using localization algorithms, providing complementary information to the authorities for intervening at an early stage.

Several strategies for source localization have been proposed in the literature [1]. These strategies have been integrated into robot systems with tracking abilities. Many of these tracking strategies have been inspired by bacteria or animal behavior using olfaction for foraging or mating: lobster, blue crabs, ants and moths provide behavioral models for odor tracking [2,3]. One of the simplest strategies consists in seeking changes in local concentration within an odor plume assuming a smooth chemical gradient in a diffusion dominated flow. However, this approach, called chemotaxis [4], is not useful in a realistic environment where fluid flow is dominated by turbulence, which can be caused by forced ventilation, temperature gradients or the presence of obstacles. Under these conditions, on the order of ten min are required to determine the time-averaged concentration with sufficient accuracy to perceive the gradient concentration [5]. In consequence, the exploration of the area of interest becomes too slow. Some other strategies additionally exploit both fluid velocity information and chemical concentration (anemotaxis [6,7]). More recent proposals based on information theory, like infotaxis [8] are based in binary detections and, information plays a role similar to that of concentration in chemotaxis. Odor patches are expected to be found only intermittently in the medium, and then information is sparse. In some cases, methods aim to estimate the gas distribution through analytical Gaussian models [9,10,11], others are focused on to create a plume mapping via hidden Markov methods [12], whereas in Farrell et al. [13] a strategy for chemical plume tracing and source location declaration is presented.

Navigation experiments aiming to find chemical sources are strongly limited by the limit of detection and selectivity of the low cost chemical sensors and even medium-priced detectors (e.g., ion mobility spectrometers). Thus, the rapid decay in the chemical concentration with increasing distance from the source can be a critical issue. Poor limits of detection result in a reduced area where the plume can effectively be detected. This is especially important in applications where the search zone has an area of several square kilometers. In such situations, it becomes very important to set the detection thresholds very close to the noise level, but this would results in a high number of false alarms and most localization algorithms would fail catastrophically. To the best of our knowledge, none of the published methods have addressed this problem. 

Additionally, in any real scenario, there could be background levels of a multitude of chemicals caused by environmental pollution. Because of limited selectivity, there will be substances which will produce interference in the detector reading, hindering the detection and localization tasks. The presence of combination of detector electronic noise and mainly interfering chemical agents result in variable background readings that may change with time and with the position of the detector. These shifts in background levels hamper the selection of an optimum threshold that is usually considered to be constant all along the area under exploration. As far as we know, this problem has not been previously tackled in the literature.

To address these two issues (threshold close to the limit of detection and presence of background levels) probabilistic approaches like plume mapping Bayesian methods appear to be a good choice. Pang and Farrell published a source-likelihood mapping approach based on Bayesian inference in 2006 [14]. The main idea behind the algorithm consists in implementing a stochastic approach for plume modeling and in estimating the most likely source position considering the sequence of detection/non-detection events and fluid flow measurements along the robot‘s trajectory. Pang’s algorithm has been tested successfully with data previously collected using an autonomous underwater vehicle [13].

However, this algorithm uses binary detection events, and no chemical concentration information is used to build the probability map, since it only considers the concentrations above a certain threshold as detection or non-detection events. Moreover, after setting the threshold level, the approach assumes that the rate of false alarms is very low. In a real scenario where background signals are present, this is only achieved when the threshold is set at a high level. However, this option seriously reduces the maximum plume detection distance. Therefore, there is a trade-off; on the one hand, the threshold needs to be set low enough (close to the sensor detection limit) if chemicals from the source are to be detected at large distances; on the other hand, the threshold needs to be high enough to prevent false alarms. So, how to set the threshold level becomes a critical issue in real environments using existing approaches, especially when the background intensity is non-uniform in the explored area.

Finally, in order to speed up the area exploration it is important that the algorithm can be extended to work with multiple robots. Recently, Kang and Li [15] have presented a novel plume tracking algorithm via multiple autonomous robots by using a leader-follower strategy, demonstrating its superiority versus a single robot algorithm, in terms of both the computational cost and the accuracy in source location. Meng et al. [3] have also studied the multi-robot problem for plume tracking in fluctuating airflow environments, showing the efficiency and robustness of the adapted ant colony optimization (ACO) algorithm over the traditional ACO algorithm. Meng remarks the importance of a proper number of robots and a well-defined cooperation mechanism, although it is not strictly necessary to track the plume to obtain a good estimation of the source location. The most likely source position is estimated during the robot's mission for arbitrary trajectories by recursively building a probability map using Bayesian inference. The problem of background estimation over the exploration area can be considered as the problem of scalar field mapping. Algorithmic approaches using mobile sensor networks have been already proposed, however they assume that the agents have communication capabilities so that their exploration paths are optimized after data fusion [16,17,18]. However, in our scenario we expect that the sensors can be mounted on vehicles that serve other tasks beyond chemical source localization. In this sense, we consider cases where there is no feedback between the chemical sensing and the agent’s trajectories.

Our main motivation for the present work is to extend the Bayesian plume source localization algorithm, previously described by Pang and Farrell [14], using the chemical concentration (instead of binary detections) and assessing its performance in simulated and real environments, where background signals may arise. Thus, Pang’s algorithm is reformulated for use with continuous analog concentration readings instead of binary detections. Moreover, the algorithm is extended to work with multiple mobile sensors. This new approach requires a probabilistic model for the background and for the plume which are described in the following sections.

The present algorithm was initially developed [19] for applications to Home Security (European project LOTUS: Localization of Threat Substances in Urban Society FP7-SEC-217925). In the considered scenario, police cars equipped with GPS and gas chemical sensors (eventually other fast analyzers like ion mobility spectrometers can be used) carry out their routine patrols while sending the sensors’ readings to a central station. Instead of moving towards the chemical source, the patrol vehicles would maintain their normal patrol routes while a centralized system is continuously analyzing the acquired signals seeking suspicious activity. 

The paper is organized as follows: Section 2.1 and Section 2.2 and shows the basics of the algorithm and the plume and background model. Section 2.3 and Section 2.4 describes the synthetic and real scenarios for the test and Section 3 reports the results and the analysis.

## 2. Materials and Methods 

### 2.1. Stochastic Models for Plume and Background

Our proposal requires making some assumptions about the dispersion of the plume (the stochastic model in Section 2.1.1) and a background model (Section 2.1.2). Given an instantaneously measured concentration *c*, it is assumed that there are two additive contributions: one due to the background ($c_{b}$) and one due to the plume ($c_{p}$), thus:

(1) $$c=c_{b}+c_{p}$$

Since we consider all these concentrations as random variables, the probability density function (PDF) of the measured concentration will be the convolution of the PDFs of the two additive terms. In the next sections, we will describe how we model the probability density of both terms. 

#### 2.1.1. Stochastic Model for Chemical Concentration Measurements 

The basis of our stochastic model [20] for the chemical plume is the analytical Gaussian plume model (GPM) [21,22]. Time-averaged plume concentration follows a Gaussian distribution lengthways to the flow direction if the time average is at least 10 min, as demonstrated in previous works [23,24,25]. This model has been widely used for its simplicity and is appropriate when dispersion is governed by atmospheric turbulence under uniform wind conditions. Atmospheric turbulence is determined by the stability of the atmosphere and the height above the surface layer [22]. The basic expression for the GPM under a continuous release is:

(2) $$\bar{c}(x,y,z)=\frac{q}{2\pi U_{a}\sigma_{y}\sigma_{z}}\exp\left(-\frac{y^{2}}{2\sigma_{y}^{2}}\right)\exp\left(-\frac{{\left(h-z\right)}^{2}}{2\sigma_{z}^{2}}\right)$$

where $\bar{c}$ is the time averaged concentration (in g/m^3^) in a location with coordinates x (downwind), y (crosswind) and z (vertical); q is the continuous source release rate or source strength (in g/s); $U_{a}$ is the mean wind speed in the downwind direction (in m/s); h is the plume height (in m); and $\sigma_{y}$, $\sigma_{z}$ are the dispersion coefficients (in m), in crosswind and vertical direction respectively, modeled as: $\sigma_{y}=a\cdot x^{b}$, $\sigma_{z}=c\cdot x^{d}$, where a, b, c and d are parameters obtained from a table [22] and their values depend on the atmospheric conditions which can be organized in six levels (from A-very unstable to F-very stable) according to the Pasquill’s stability classes [26]. In Equation (2), the resulting concentration distribution is due to the transport of chemicals by advection (due to the mean wind speed), due to concentration gradients within the plume width (lateral dispersion due to diffusion, but also turbulent mixing) and due to plume meandering. The decay of mean concentration is exponential, thus concentration levels below the sensor detection limit are very quickly achieved. This issue makes the setting of the threshold level critical; especially, if the source should be detected far from the release point. 

The GPM considers the time-averaged characteristics of a plume dispersed in a turbulent flow, but the sensors will be responding to the instantaneous plume characteristics (here we assume that the chemical sensor dynamics are much faster than 10 min, which is usually the case). For short time-scale studies, the chemical puff movement can be modeled as a random walk (because of transversal velocity fluctuation) overlapped on the downflow advection (because of mean velocity) [27]. However, we propose an alternative approach, for which an additional component needs to be added to the GPM to model the unpredictable and random fluctuations in concentration due to turbulent stirring and plume meandering. Yee et al. [28,29,30] have carried out empirical studies on plume statistics in urban areas using scale fluid models in a variety of plume conditions and urban geometries. Their results prove that instantaneous concentration fluctuations fit very well the clipped-gamma PDF over a very wide range of atmospheric conditions and at several receptor positions [30]. The clipped-gamma distribution (CGD) is defined in terms of four parameters γ, $k^{*}$, s and λ as [28]:

(3) $$f(\hat{c})={\left(\frac{\hat{c}+\lambda }{s}\right)}^{k*-1}\frac{\exp\left(-(\hat{c}+\lambda )/s\right)}{s\Gamma (k*)}+(1-\gamma )\delta (\hat{c})$$

where $\hat{c}$ is the instantaneous concentration, $\Gamma \left(\hat{c}\right)$ is the gamma function, $\delta \left(\hat{c}\right)$ is the Dirac delta function and λ, $k^{*}$ and s are the shift, shape and scale parameters, respectively. The total PDF is composed of a mixed fluid part due to in-plume mixing of eddies containing the target substance (the first term on right-hand side), and an unmixed ambient fluid part (the second term on right-hand side) caused by plume meandering which produces intermittent periods of zero concentration for a fraction of time (1 − *γ*), being *γ* the intermittency factor. Although Equation (3) is specified in terms of four parameters, it can be uniquely modeled by the mean (M) and the standard deviation (SD) of a series of readings [28]. There is a simple relation among M, SD, and the plume intermittency; thus, *γ* is determined as:

(4) $$\gamma =\gamma (k*,s,\lambda )=\frac{\Gamma \left(k*;\lambda /s\right)}{\Gamma (k*)}=\min\left(1,\frac{3}{K_{\mathrm{int}}{}^{2}+1}\right)$$

where Γ(v;c) corresponds to the incomplete gamma function and $K_{int}$ is the ratio between the SD and the mean of the series of readings at a fixed position. For a specified value of $K_{int}$, the parameters λ, $k^{*}$ and s can be obtained solving a set of transcendental equations, thus making Equation (3) totally defined. The details for computing these parameters are given in [28].

While the mean concentration decreases rapidly in the downwind direction (see Equation (2)), the magnitude of the fluctuations decreases even more rapidly. As described by Webster [5], $K_{int}$ is estimated to decrease as $x^{\theta }$ (where x is the distance from the source and θ<0), being roughly $K_{0}$ times the mean time-averaged concentration at a certain distance $x_{0}$ from the source. Therefore, this parameter can be modeled as:

(5) $$K_{\mathrm{int}}=K_{0}\cdot {\left(\frac{x}{x_{0}}\right)}^{\theta }$$

To model the instantaneous concentrations due to a chemical plume at a certain distance from the source, the clipped-gamma distribution is used (Equation (3)). The mean value M of the series of concentrations due to the plume is related to the GPM (Equation (2)) and the SD can be modeled as:

(6) $$\sigma_{SD}=K_{0}\cdot {\left(\frac{x}{x_{0}}\right)}^{\theta }M$$

Since the PDF depends on the mean and the SD, and these parameters depend on the distance from the source, the PDF of the instantaneous readings contains information about the relative position between the sensor and the source. Since concentrations fluctuations (intermittencies) decrease faster than the mean value with the downwind distance to the source, the plume becomes homogeneous faster than the mean concentration dilutes [28,31].

Additionally, previous literature has characterized the power spectral density (PSD) of concentration readings within a dispersing plume. We will follow the model described by Jones et al. [31].

#### 2.1.2. Stochastic Background Model

In real scenarios such as residential areas or urban environments, pollution or interfering substances are expected to be found. This problem becomes even more serious due to the common use of partially selective sensors such as Metal Oxide Sensors (MOXs), or Photoionization detectors (PIDs).

Moreover, meteorological conditions (wind conditions and atmospheric stability) could change within a timescale of several hours, or there might be changes in polluting emissions due to day-night cycles of human activity (including motor vehicles or factories) [32,33,34]. Because of these reasons the background can be considered to change slowly. 

As it has been previously described [5], the dispersion of a chemical plume in a turbulent flow shows a highly intermittent nature with background or zero concentration for long periods of time separated by high peaks of concentration. This behavior will help us to estimate the background model using robust measures that reject the plume peaks.

For the implementation of the proposed algorithm the exploration area is divided into a uniform grid of rectangular cells. The algorithm estimates the background probability distribution at each cell of the grid. The background is modelled as a Gamma PDF where the standard deviation is smaller than the mean. In those conditions the Gamma function resembles a Gaussian PDF but defined only for positive values of the random variable concentration. We will consider that the PSD of the background is bandlimited white noise.

### 2.2. Estimation of the Models From the Concentration Readings 

#### 2.2.1. Bayesian Estimation of the Likelihood Map for Chemical Source Presence

A summary of the notation used for the description of the algorithm can be found in Table A1. The search area contains $N_{c}$ rectangular cells of size $L_{x}\cdot L_{y}$ each, where $L_{x}$ and $L_{y}$ are the cell lengths in the x and y directions of the grid map, respectively. The size of this cells is a trade-off between the spatial resolution of the algorithm and the computational cost. 

Let $0\leq \alpha_{i}^{'}\leq 1$ represent the probability that the chemical source is in cell i. It is assumed that the search area contains exactly one source, hence $\overset{N_{c}}{\underset{i=1}{\sum }}\alpha_{i}^{'}=1$. It is assumed that the prior information on the potential existence of a chemical source is given by previous intelligence research. We do not consider in this formalism the case where there is uncertainty on the presence or not of a chemical source. 

Initially (at $t=t_{0}$), if no information about the source location is available, all cells are initialized to be equally likely to contain the chemical source: αi'(t0)=(1Nc); $\forall i\in \left[1,N_{c}\right]$.

Given that we measure a concentration at time $t_{k}$ in cell j, i.e., $c_{j}$ refers to a concentration reading in cell j, we can calculate the source probability map based on this single reading. A Bayesian approach is used to determine if the main contributor to the measurement is the background or the presence of a plume patch. The posterior probability for the presence of a plume, given the measurement, is calculated using Bayes’ theorem:

(7) $$P(A|c_{j})=\frac{P(c_{j}|A)\cdot P(A)}{P(c_{j})}$$

where A corresponds to the event “the concentration reading was caused by an emitting source upstream (plus a background level)”. To infer where the source is located, the posterior probability of a source emitting (left-hand term in Equation (7)) can be further decomposed into the probability of that source being located at each cell. Again, using the Bayes’ Theorem:

(8) $$P(A_{i}|c_{j})=\frac{P(c_{j}|A_{i})\cdot P(A_{i})}{P(c_{j}|A_{i})\cdot P(A_{i})+P(c_{j}|{\bar{A}}_{i})\cdot \left[1-P(A_{i})\right]}$$

where $\bar{A}$ means “the concentration reading was caused only by background levels and not by an emitting source”, subindex i refers to a source located in cell i and subindex j refers to the current cell j where the measurement was taken. 

Since we consider that a background of interfering substances is always present, in the absence of plume only the background component $\left(c_{b}\right)$ is present; in the presence of plume, both components ($c_{b}$ and $c_{p}$) are present and the concentrations are modeled by the convolution of the plume PDF and the background PDF. However, even in the presence of the plume $c_{p}$ may be zero due to plume intermittency. The PDF of the concentration component due to the source is modeled using the GPM for the means, and SD from Equation (6) considering the relative position of cell i (potential source location) and cell j (location of the sensor). 

Taking these considerations into account, the previous probabilities have the following interpretation: $P\left(A_{i}\right)$ is the prior probability of the presence of a source at the cell i, $P\left(c_{j}|A_{i}\right)$ is the probability that the measurement at cell j is due to addition of the background at cell j and a plume due to a source at cell i, and it is obtained by the convolution between the PDF of the plume and the PDF of the background at cell j. $P\left(c_{j}|{\bar{A}}_{i}\right)$ is the probability that the measurement of chemicals at cell j is due to the current background at cell j, and it is obtained from the PDF of the background at cell j. We define $S_{ij}\left(t_{k}\right)\equiv P\left(A_{i}|c_{j}\right)$ as the probability of having a source in cell i given that a certain amount of chemical was measured at cell j at time $t_{k}$. 

However, in this approach where we consider each cell independently, $\overset{N_{c}}{\underset{i=1}{\sum }}S_{ij}\left(t_{k}\right)=1$ is not guaranteed. Therefore, the result is normalized to ensure the total probability is 1 when individual cell probabilities are added up. The nomenclature is henceforth the same as that used in [14]:

(9) $$\beta_{ij}(t_{k})=\frac{S_{ij}(t_{k})}{\sum_{i=1}^{N_{c}}\left[S_{ij}(t_{k})\right]}$$

Now $\overset{N_{c}}{\underset{i=1}{\sum }}\beta_{ij}\left(t_{k}\right)=1$ is guaranteed and $\beta_{ij}\left(t_{k}\right)$ calculated over all cells ($i=1,...,N_{c}$) gives the source probability map at time $t_{k}$ based on a single measured concentration at cell.

Using Bayesian theory [20] and following the same procedure described in [14], each new measurement can be incorporated recursively to update the source probability map. 

${\alpha^{\prime }}_{ij}\left(t_{k}\right)=P\left(A_{i}|B\left(t_{k}\right)\right)$ is defined as the probability of cell i containing the source, given the sequence of concentrations $B\left(t_{k}\right)$ along the trajectory of the mobile sensors up to time $t_{k}$. Defining $P\left(A_{i}|D_{j}\left(t_{k}\right)\right)=\beta_{ij}\left(t_{k}\right)$, where $D_{j}\left(t_{k}\right)$ is the measured concentration at time $t_{k}$; $P\left(A_{i}|B\left(t_{k}\right)\right)$ is computed from $B\left(t_{k-1}\right)$ and $D_{j}\left(t_{k}\right)$, which are supposed to be independent events, finally obtaining:

(10) $$\alpha^{'}{}_{ij}(t_{k})=N_{c}\cdot \alpha {'}_{ij}(t_{k-1})\cdot \beta_{ij}(t_{k})$$

where, if ${\alpha^{\prime }}_{ij}\left(t_{k}\right)$ is computed over all cells ($i=1,...,N_{c}$), an updated source probability map is obtained recursively. 

Independently of the number of mobile sensors, the only information required by the algorithm is: the position where the measurement was obtained, the chemical concentration reading and the fluid flow measurement. The extension to multiple mobile sensors is as follows. We just build an integrated sequence of readings by addressing in a circular manner the set of mobile sensors. Then we send this sequence of measurements to the original algorithm. In other words, we do not fuse a posteriori maps built from individual sensors. We fuse the sequence of measurements at the input of the estimation algorithm.

#### 2.2.2. Background Estimation

Background stochastic model is built from the robust estimation of the means and the dispersion to reject the effect of the intermittencies. We use the median and the median absolute deviation (MAD) to build the background probability density model [35]. These parameters are estimated over a buffer of the last 50 measurements. These measurements can be spread out in several cells depending on the speed of the robot (typically less than 10). This is not a problem if the spatial variation of the background is sufficiently smooth. 

To allow for the model to adapt to a slowly changing background the parameters of the model (mean and standard deviation) are filtered with an exponential moving average. This filter weights the current estimation of the background with the old one depending on the time distance. In this way, the system is able to forget the old values and adapt to new ones. 

### 2.3. Description of the Synthetic Test Cases

#### 2.3.1. Synthetic Scenario Description and Simulation

For realistic simulations, the scenario envisioned considers atmospheric plumes in an urban area encompassing hundreds of thousands of square meters containing only one chemical source. The sensors located in vehicles can transmit their current position in the grid (e.g., using a GPS sensor) together with the chemical sensor readings, and they will explore the search area by moving across the cells performing random exploration. It is assumed that each vehicle mounts a single sensor. It is considered that the main task of the vehicles is not that of tracking the plume; but patrolling a certain area and simultaneously updating a probability map for the source location using the available information. 

**Figure 1:**
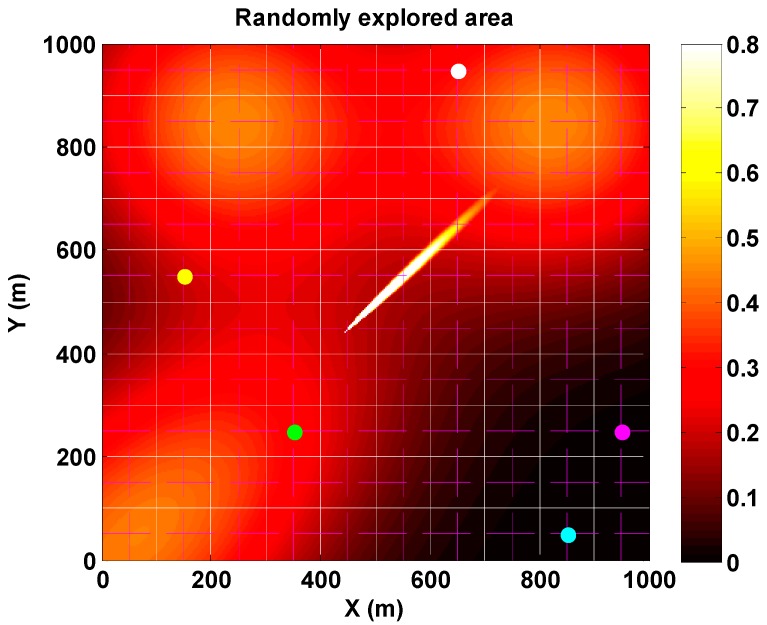
Randomly explored area (1 km × 1 km) using five sensors (colored dots). The continuous white lines (-) define the cell boundaries. The sensors move in horizontal or vertical direction from the center of one cell to the center of a neighbor cell. The source is located at (440, 440) meters at cell (5, 5). Mean wind speed is set to 2.5 m/s and wind direction to 45°. Colored background refers to probability map at a particular time step.

The exploration arena in the synthetic case is as follows. In order to test both algorithms, a synthetic scenario (grid size of 1 km × 1 km) is generated. The area is divided into cells of size 100 m × 100 m. A square sub-grid of lanes (100 m separation between lanes) is interlaced over the main grid (Figure 1). The sensors will randomly explore the area over this last sub-grid of lanes which simulate streets within an urban environment (Manhattan style). Using this configuration, the movement of the sensors is constrained to certain lanes over the main grid. A clandestine laboratory for home-made explosive production emits explosive precursors. In this scenario, we consider that a chemical source emitting with a source strength q=2.90 g/s, is placed in the grid with the source at the position (440 m, 440 m), which corresponds to coordinates (5, 5) on the rectangular grid. We consider that the main dispersed substance is acetone (molecular weight: 58.08 g/mol) at one atmosphere pressure and 25 °C. This Gaussian plume distribution has been generated from Equation (2) with the plume being dispersed in a 2D plane at the same height as the sensors (z=h=2 m). It is assumed that there is no deposition of the substance on surfaces. In the simulations, the wind field is constant with the wind speed at $U_{a}=2.5$ m/s and the wind direction at 45°. The dispersion coefficients $\sigma_{y}$ and $\sigma_{z}$ depend on wind conditions and atmospheric stability which has been set to neutral (‘D‘ on the Pasquill-Gifford scale [26]). Moreover, a mean background distribution is deployed over the area with a different mean level in each cell and with SD equal to 60% of the mean value in all cells (based on our own recorded data using a PID sensor measuring in Barcelona outdoors over a period of several hours). 

Five mobile sensors with a constant velocity of 15 km/h sense the area continuously. The sampling period of the sensors is set to 3 s, the detection limit to 0.1 ppm and the sensor resolution to 0.01 ppm, which are realistic values for PID technologies. We assume that the response time of the sensor is much faster than the typical 10 min time-average considered in the Gaussian Plume Model (Equation (2)). The total simulation time was set to 300 min.

The mean background level is different in each cell, but is stationary over time. Series of concentration fluctuations are generated in each cell considering the wind field created, the atmospheric conditions and the background. The stochastic model of the plume concentrations has been already described in Section 2.1.1. However, here we give some additional technical details concerning the practical implementation.

Since we have defined the PDF and the PSD which will be used to model concentration fluctuations, we use the percentile transformation method (PTM) described by Papoulis [36] to generate a series of concentration fluctuations with the desired PDF and PSD.

Specifically, the procedure to generate realistic plume readings consists of the following steps: (i) generate a time series of Gaussian white noise, (ii) filter the previous time series with the designed FIR filter to achieve the desired PSD, (iii) and apply the PTM. This method is based on the following expression:

(11) $$c_{i}=F_{c}^{-1}(F_{z}(z_{i}))$$

where $z_{i}$ is a random sequence of Gaussian white noise having the desired PSD with cumulative distribution function (CDF) $F_{z}\left(z\right);\;c_{i}$ is the sequence of realistic readings in the cell with CDF $F_{c}\left(c\right)$. This CDF corresponds to the clipped-Gamma CDF (Equation (3)):

(12) $$F(c)\equiv \mathrm{Pr}(C\leq c)=\int_{0^{-}}^{c}f(c^{'})dc^{'}=1-\frac{\Gamma (k^{*};(c+\lambda )/s)}{\Gamma (k^{*})}$$

and $F_{c}^{-1}$ is the inverse clipped-Gamma CDF. The clipped-Gamma PDF (and its CDF) depends on the distance to the source. Its two parameters, the mean and the SD, are obtained from Equation (2) and Equation (6), respectively. Subsequently, these parameters are used to compute γ, $k^{*}$, s and λ, as explained in detail in [28]. 

Background concentrations are simulated as white noise with Gamma PDF. The background concentrations in each cell are added to the time-series of plume readings to obtain the final concentration readings at each cell. 

**Figure 2:**
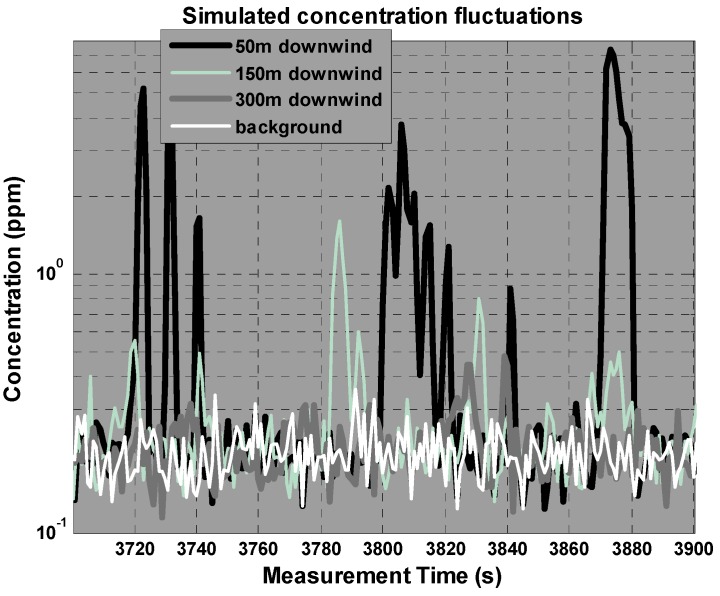
Simulated concentration fluctuations (under the specified conditions) at different fixed positions downwind from the source over a certain background level.

An example of the concentration signals delivered by the sensors in this simulation scenario is shown in Figure 2. It can be observed that the SD of the fluctuations decreases faster than the mean concentration, making it difficult to differentiate between plume and background far from the source.

In the binary-based approach, it is assumed that the ratio of false alarms is very low, but the ratio of missed detection can potentially be very high, thus we define μ = 0.3 (70% missed detections). However, the value of this parameter was not defined in the original work [14]. 

In the concentration-based approach, the source strength (q) should be initially guessed (this is typically done using previous information about the type of chemical source under investigation: clandestine lab, industrial toxic emissions, pollution, etc.). However, since it is almost impossible to guess this parameter with accuracy, we have studied the sensitivity of the algorithm to errors in this parameter. We scanned the guessed source strength two orders of magnitude around the central exact value. For the simulation studies presented in this work, the parameters used in Equation (6) are: $K_{0}=2.5$, $x_{0}=50\;m$ and θ=− 0.75, taking into account previous studies [5]. 

#### 2.3.2. Synthetic Test Case 1: Behavior of the Binary Detector Algorithm Depending on the Background Level and Detector Threshold

The first simulation aimed to characterize binary-based approach when changing the concentration threshold that fires the detector signal. In order to investigate the robustness of the method against chemical noise, two spatially uniform background levels were considered (mean values: 0.05 ppm and 0.45 ppm). In both cases the standard deviation was 1/5 of the mean value. 

A background level is low or high depending on the source strength; therefore, studying the case where the background is low is equivalent to saying that the source is potent, and studying the case where the background is high is equivalent to saying that the source is weak.

Ten random sensor trajectories were simulated for each threshold level in the binary detector (30 values in the linear range between 0.05 ppm and 3 ppm). For each trajectory, the probability at the real source location is assigned by the algorithm after 300 min of exploration time. We took as a figure of merit the mean probability averaging over all trajectories. This figure of merit will be plotted against the concentration threshold. For the binary case, this simulation will determine the optimum threshold for the detector given a certain chemical power source.

#### 2.3.3. Synthetic Test Case 2: Accuracy in the Estimation of the Background Level and the Expected Position of the Chemical Source

Here, both algorithms (binary and analog) are compared and share the same 10 random trajectories. In this second case, a non-uniform background is used. Results will show the probability evolution at real source location as the exploration time increases. Moreover, the probability maps provided by both approaches after 300 min of exploration time can be compared as well. 

To assess the accuracy of a given probability map we compute the expected value of distance to the true source position:

(13) $$E\left[r-r_{0}\right]=\sum_{i=1}^{N_{c}}p\left(r_{i}\right)\cdot \left(r_{i}-r_{0}\right)$$

where r is the estimated position of the source, $r_{0}$ is the real source position and $p\left(r_{i}\right)$ represents the probability of the source being located in cell i.

Based on this metric we calculate the root mean squared error ($RMSE_{p}$) of the distance from the expected source position to the real source location:

(14) $$RMSE_{p}=\sqrt{\left(E\left[\|r-r_{0}{\|}^{2}\right]\right)}=\sqrt{err_{x}^{2}+err_{y}^{2}}$$

Alternatively, for a richer description we can decompose the total error in both coordinates: $err_{x}$ and $err_{y}$. Alternative, though less informative, figures of merit are the Euclidean distance (D) between the maximum of the likelihood map and the real source position, and the probability at the real position. 

The mean background map recovered for the concentration-based algorithm is quantitatively compared to the designed background distribution by using the root mean squared error ($RMSE_{b}$) as a figure of merit:

(15) $$RMSE_{b}=\sqrt{\frac{\sum_{i=1}^{Nc}{\left(B_{i}-\hat{B_{i}}\right)}^{2}}{N_{c}}}$$

where $B_{i}$ is the designed mean background level at cell i; and ${\hat{B}}_{i}$ is the estimated mean background level. Results are obtained for two background distributions (maximum mean values: 0.05 ppm and 0.45 ppm). Both background concentration maps were the same except for a scale factor. The binary-based approach was tested using the optimum threshold level determined from case 1 and the concentration-based approach, using the exact source strength. 

#### 2.3.4. Synthetic Case 3: Influence of the Source Strength on the Concentration Based Algorithm

Finally, the third simulation shows the influence of the source strength in the overall performance of the concentration-based approach. Since it is difficult to know the source strength in advance, the performance of the algorithm has been assessed assuming different source strengths across more than two orders of magnitude in the range between 0.1 g/s and 30 g/s (the real value in the scenario simulator being 2.90 g/s). Results are shown for the same background distributions as in case 2 and for 10 random trajectories.

### 2.4. Scenario, Chemical Source Emission and Autonomous Vehicle Description for Real Experiments

For real experiments, all measurements were performed within an exploration arena built with polystyrene panels. Dimensions of the tunnel were 5 m × 3.5 m × 1.8 m (length × width × height). One fan was introducing air into the room and, on the opposite wall, three fans extracted air from the room. All of them were installed at a height of 0.9 m above the floor and generated a highly a turbulent airflow. Fans used were helical-wall type with a diameter of 30 cm and a maximum speed of 1300 rpm. 

The supply of volatile acetone was carried out employing two 10-mL syringes. These syringes were filled with liquid acetone and assembled on a syringe pump KDS-200 (KD Scientific, Holliston, MA, USA) which was programmed to deliver a controlled liquid flow of 150 µL/min during 60 min. Thus, the liquid acetone was falling at a constant rate over a plate heated to a temperature sufficient for the acetone to be evaporated immediately. The source emission of volatile acetone was placed at one side of the exploration arena just in front of the fan that introduced air in the tunnel. Algorithms for the generation of the probability maps operated over a grid of $N_{c}=70$ cells with dimensions 0.5 m × 0.5 m each one.

Input data for the algorithms were registered by an autonomous vehicle which was constructed on a metal structure where different components were assembled, such as: two DC motors that allow mobility of the vehicle, an UTM-30LX USB laser range finder sensor (HOKUYO, Osaka, Japan) for vehicle auto-location and navigation; and a Windsonic RS232 anemometer (Gill Instruments, Lymington, England) to register the wind speed and direction. The autonomous vehicle was also equipped with a photo ionization detector (PID, ppbRAE 3000, RAE Systems, San Jose, CA, USA) for measurement of the concentration of volatile compounds. All these components were controlled by an onboard computer. The vehicle could operate autonomously sampling its relative position, the wind speed and direction, and the volatile compound concentration at one measurement every second approximately. The vehicle was programmed to move in a straight line until a wall is found, then the vehicle rotates a random angle, and a new straight trajectory is described until a new wall is found. The vehicle speed was set to 0.2 m/s. Images of the robot, the exploration room and the chemical source can be found in the supplementary materials. 

## 3. Results and Discussion

In this section, we will introduce the results for the three cases already described, as well as the results for the real experiments. Finally, we will discuss the computational cost of the presented algorithm. Data are presented as 50th [5th–95th] percentiles unless otherwise specified.

### 3.1. Results of Algorithms Evaluation for Synthetic Experiments

#### 3.1.1. Synthetic Case 1

**Figure 3:**
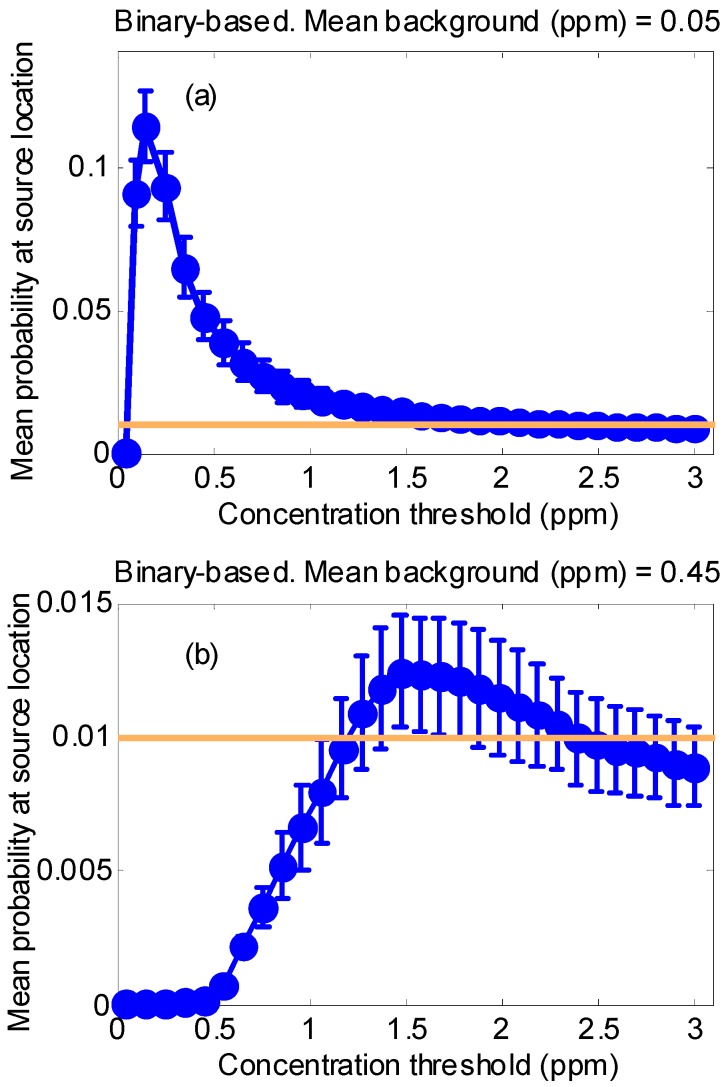
Dependency of the assigned mean probability (averaged over 10 different random trajectories) with the concentration threshold at source location by the binary-based approach. (**a**) Low background level (mean = 0.05 ppm and SD = 0.03 ppm). (**b**) High background level (mean = 0.45 ppm and SD = 0.27 ppm). The orange line shows the initial equiprobable value ($1/N_{c}$) assigned to every cell.

The overall performance of the binary-based approach as the concentration threshold is changed is studied in synthetic case 1. The results for two different background levels: the former with a mean value of 0.05 ppm (Figure 3a) and the latter with a mean value of 0.45 ppm (Figure 3b) are shown. The mean probability assigned by the algorithm at the real source location is depicted as a function of the concentration threshold. It can be seen in the figure that there is a different optimal concentration threshold depending on the background level. As expected, the optimum value is shifted to higher thresholds as the background level is increased. These optimal values have been found to be 0.15 ppm and 1.48 ppm, respectively. Relative to the background level, setting the threshold too low produces a high ratio of false alarms leading the algorithm to failure; on the other hand, setting the threshold too high could lead to an increase in false negatives with abnormal concentrations considered as non-detection events and a consequent worsening of the overall performance. This can be critical if the source to be detected is weak –this being equivalent to the case with a high background level. In Figure 3b, it is observed that setting the threshold either too low or too high causes the algorithm to fail, since the probability assigned to the real source location is below the equiprobable value of $1/N_{c}$ assigned initially to every cell.

The main problem using the binary-based approach is that the threshold needs to be set arbitrarily if no information about the background is available and this background can be different in various areas within the exploration zone. A priori, we do not know whether the threshold is too high or too low, but even if we knew this, the background could evolve over space and time and the threshold would need to be adjusted continuously. The concentration-based algorithm removes the necessity of any threshold because, instead of adjusting the threshold level, our approach builds a background model for each cell. This background model allows us to distinguish between the background and the plume without using any threshold and is updated recursively.

#### 3.1.2. Synthetic Case 2

**Figure 4:**
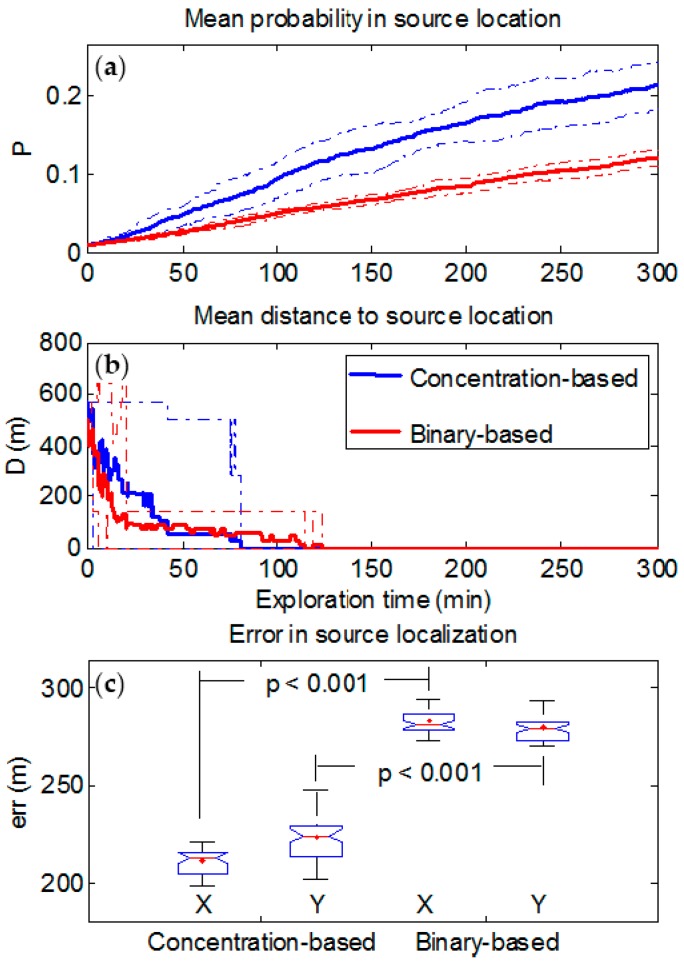
Performance of binary-based and concentration-based algorithms in source localization with a maximum mean background level of 0.05 ppm. (**a**) Mean probability averaged over the ten random trajectories (thick solid line) and confidence intervals within two standard deviations (thin dashed lines). (**b**) Mean Euclidean distance D between the cell with the highest probability value and the real source location (thick solid line) and both the maximum and the minimum values (thin dashed lines). (**c**) error in source localization in both X and Y directions at the end of exploration time; *p*-values between both approaches by using the Wilcoxon rank test have been indicated.

We will first analyze the results for the case of a weak interfering background having a maximum mean value of 0.05 ppm. Figure 4 shows the performance of the binary-based and concentration-based algorithms in source localization by considering the evolution of the figures of merit. Mean probability (averaged over all trajectories) increases throughout the exploration time (Figure 4a) in both approaches. 

The Wilcoxon nonparametric rank test (not shown) was applied at each time step to test the null hypothesis of no difference between the median of both populations (i.e., those formed by the 10 probabilities values in each of the two algorithms). Approximately 15 min after the starting time, the increase in probabilities for the concentration-based approach become statistically significant (*p* < 0.05) in comparison with the probabilities for the binary-based approach which did not change until the end of exploration time. Figure 4b shows the mean Euclidean distance D between the cell with the highest probability value and the real source location. For both approaches, mean distance decreases as the exploration time increases. In average, the binary-based approach converges to the real source location slower (125 min. approximately) than the concentration-based one (80 min approximately), and with lower probability value as it can be observed in Figure 4-top. Moreover, the errors in source localization algorithms in both X and Y directions of the grid (Figure 4-bottom) show that the concentration-based algorithm performs significantly better (*p* < 0.001), *err* = 213 [199 − 222] m and *err* = 225 [202 − 248] m for X and Y directions respectively, in comparison with the binary approach (281 [274 − 294] and 279 [271 − 294]), even though the binary-based algorithm has been tested with the optimum concentration threshold (0.15 ppm).

**Figure 5:**
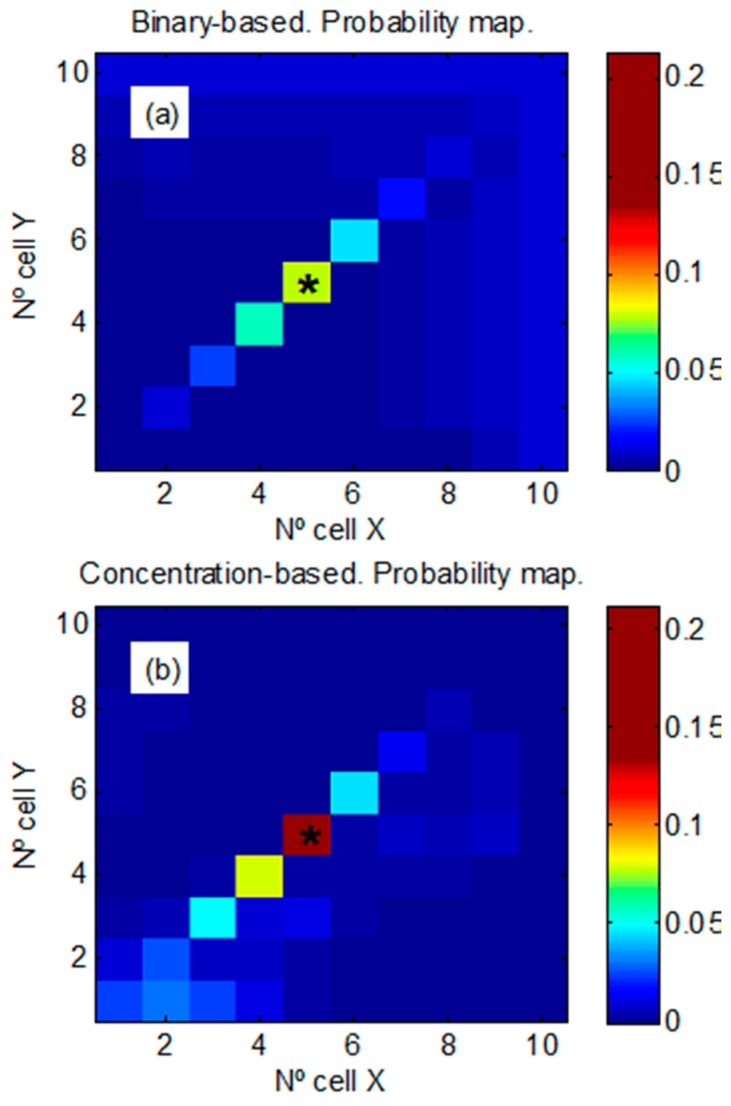
Mean probability maps (averaged over all trajectories) after 300 min of random exploration with a maximum mean background level of 0.05 ppm. Source location at (5, 5) is indicated with an asterisk (*). (**a**) Binary-based approach. (**b**) Concentration-based approach.

Figure 5 shows the comparison between the mean probability maps (averaged over all trajectories) provided by both algorithms after 300 min of random exploration. The probability assigned to the real source location is higher using the concentration-based approach (P = 0.215 [0.156 − 0.261] in Figure 5b) as compared to the binary-based approach (P = 0.124 [0.102 − 0.131] in Figure 5a). Moreover, the probability is spread among a lower number of cells in the wind direction.

The estimated background distribution (not shown) by the concentration-based approach is similar to the designed one after 300 min of random exploration, $RMSE_{b}$ = 0.010 [0.009 − 0.012] m. Moreover, it was observed that after 50 min of exploration time the $RMSE_{b}$ values remained approximately constant.

**Figure 6:**
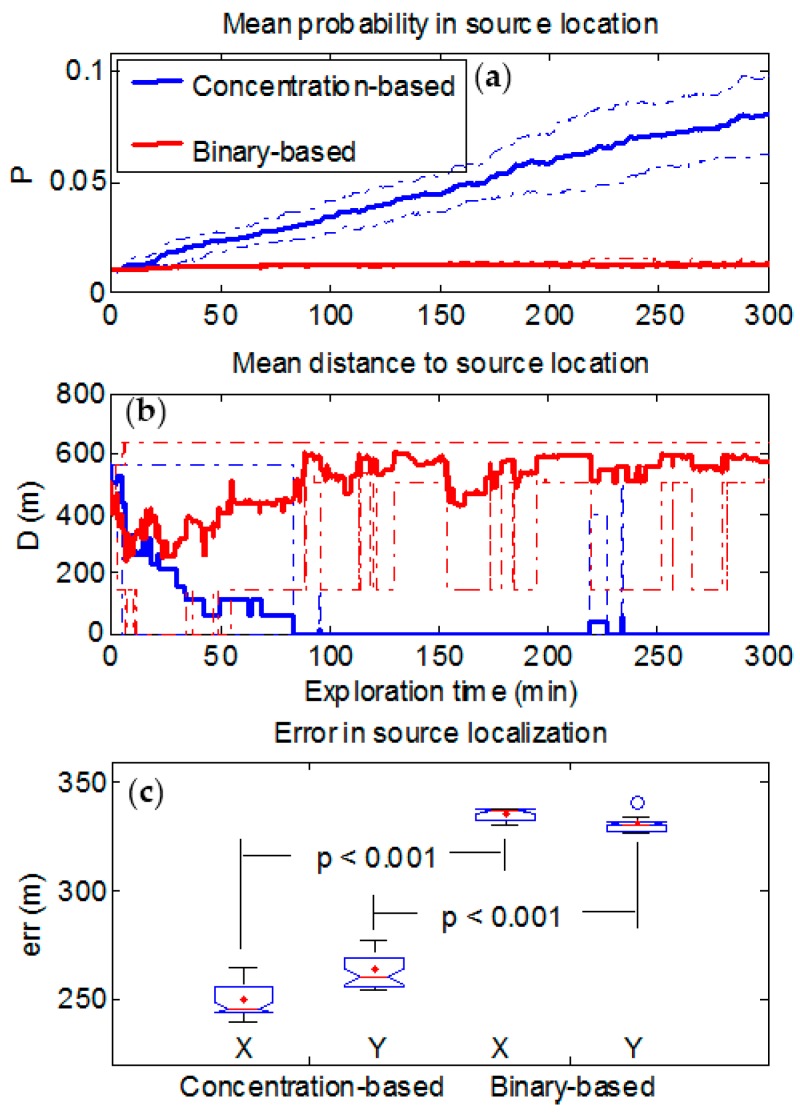
Performance of binary-based and concentration-based algorithms in source localization with a maximum mean background level of 0.45 ppm. (**a**) Mean probability averaged over the ten random trajectories (thick solid line) and confidence intervals within two standard deviations (thin dashed lines). (**b**) Mean Euclidean distance D between the cell with the highest probability value and the real source location (thick solid line) and both the maximum and the minimum values (thin dashed lines). (**c**) error in source localization in both X and Y directions at the end of exploration time; *p*-values between both approaches by using the Wilcoxon rank test have been indicated.

With a high background level (0.45 ppm), which is the same as saying that the source strength is small compared to the background, Figure 6 shows similar results to those shown in Figure 4. However, now the differences between the performances of both approaches are higher. Mean probability at real source location (Figure 6a) for the concentration-based approach increases as exploration time increases, but more slowly compared to Figure 4a. In contrast to that observed in Figure 4a for the binary-based approach, the mean probability is lower and remains approximately constant throughout the exploration time. Here, the Wilcoxon test is also used to assess the statistical difference between both approaches. Differences observed become statistically significant (*p* < 0.05) after 15 min of exploration approximately. Moreover, it is observed in Figure 6b that the mean distance for the binary-based algorithm does not converge to the real source location. The errors accounted (Figure 6c) in both the X (*err* = 246 [239 − 265] m) and the Y (*err* = 260 [254 − 277] m) directions are higher than the case of a low background and confirm that the performance of the concentration-based approach is significantly more robust than the binary-based (337 [331 − 338] m and 331 [326 − 341] m for X and Y directions respectively) in the presence of a high background level, although the latter has been tested with its optimum concentration threshold.

The binary-based approach performs well under the assumption that no false alarms arise. This is shown in Figure 4 where, due to a low background level, the number of missed detections and false alarms arising from the background are small, and the binary-based algorithm performs slightly worse compared to the concentration-based approach if the optimum threshold can be identified. Nevertheless, such an assumption is far from the truth in a real scenario where pollution and interfering substances are expected to be found. Additionally, this can also be the case when the source to be detected is weak. This is shown in Figure 6 where, due to a high background level (or weak source), the number of false alarms is higher, thus forcing setting the threshold higher, which leads in turn to an increase in the number of missed detections and worsens dramatically the performance of the binary-based approach.

**Figure 7:**
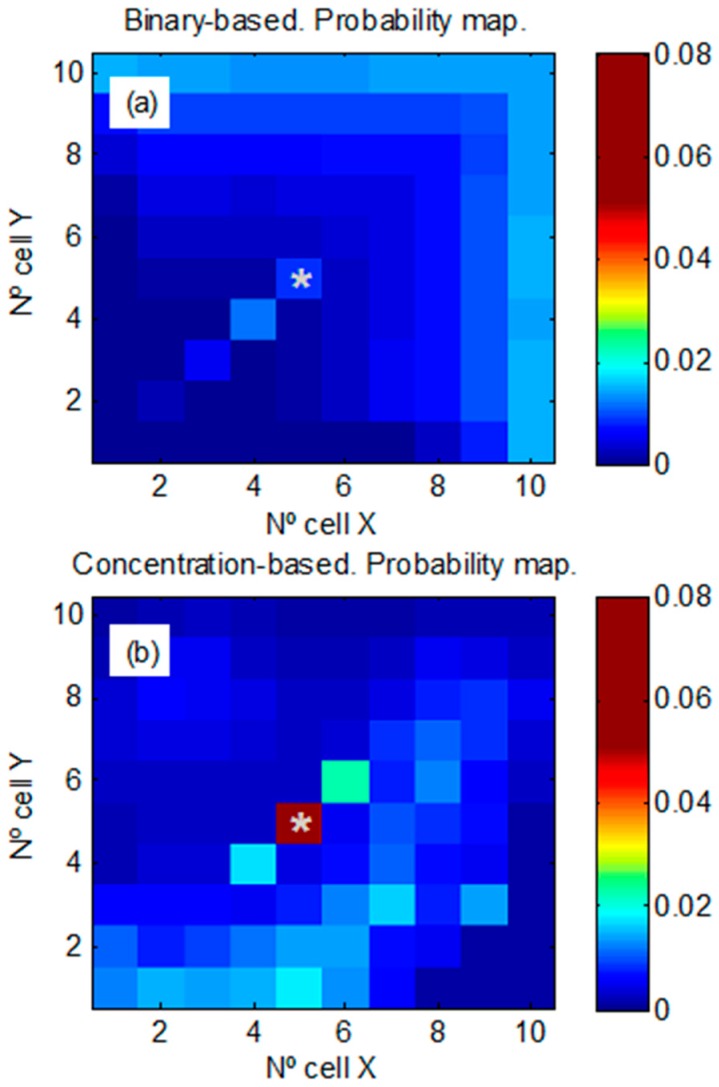
Mean probability maps (averaged over all trajectories) after 300 min of random exploration with a maximum mean background level of 0.45 ppm. Source location at (5, 5) is indicated with an asterisk. (**a**) Binary-based approach. (**b**) Concentration-based approach.

Figure 7 shows the probability maps obtained with a high background level. It is seen that the threshold is very high, which minimizes the number of false alarms but increases the number of missed detections, thus the binary-based estimation is very uncertain at the source location (Figure 7a). Therefore, the probability is spread over the cells in the grid, decreasing the probability at real source location (P = 0.013 [0.010 − 0.014]). Figure 7b shows the robustness of our algorithm which minimizes false alarms and missed detections. The concentration-based algorithm tends to increase the probability at the real source location while the vehicles are performing random exploration. After 300 min of random exploration, the probability assigned to real source location was P = 0.083 [0.047 − 0.101]. In this approach, false alarms arising from the background can correctly be assigned lower weights in the probability calculations because the algorithm has created a background model per each cell and a dispersion model for the plume. Additionally, these models allow minimizing the number of missed detections.

Concerning the estimation of the background distribution, the concentration-based approach was also able to recover it properly, $RMSE_{b}$ = 0.089 [0.080 − 0.100] m. Like in the case of a low background, the $RMSE_{b}$ remained approximately constant after 50 min, albeit, it was slightly higher.

#### 3.1.3. Synthetic Case 3

**Figure 8:**
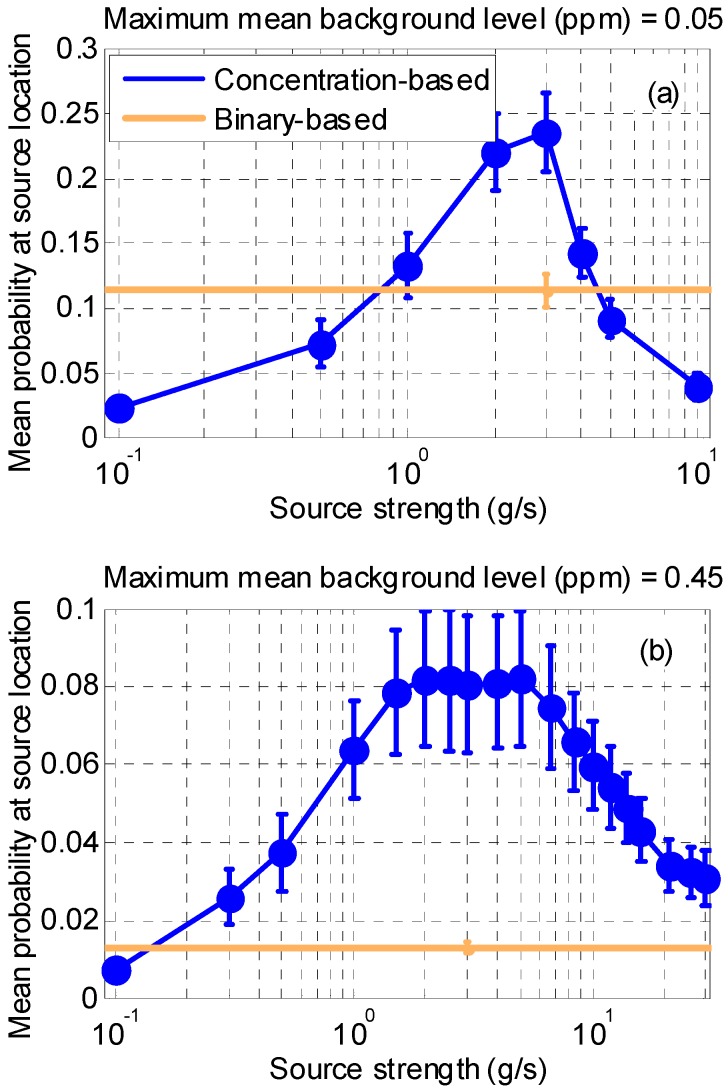
Mean probability (averaged over 10 trajectories) at real source location after 300 min of random exploration as a function of the source strength assumed by the concentration-based approach. Error bars show confidence levels within two standard deviations. (**a**) Results with a maximum mean background level of 0.05 ppm. (**b**) Results with a maximum mean background level of 0.45 ppm.

The results concerning the sensitivity to the correct estimation of the chemical source strength are displayed in Figure 8. Results show a decrease in the overall performance as the assumed source strength deviates from the real value (2.90 g/s). It is observed (Figure 8a) that the selection of the source strength becomes more critical when the background level is low (or the source is potent compared to this background), but, if the source strength could be estimated with errors (to within at most a factor of 4), the concentration-based approach performs much better. When the source to be detected is weak (or the background is comparatively high, Figure 8b), the selection of the source strength is not so critical in the range studied. It can be observed that the concentration-based approach is more robust against false alarms and missed detections (even for source strength estimated with errors larger than two orders of magnitude), as compared to the binary-based approach which performs badly even setting its optimum threshold.

It is important to say that, although the binary-based algorithm works without assuming explicitly any source strength, setting an optimum threshold is only possible when collecting real measurements which implicitly contain information about the source strength and the background. However, this optimum threshold might be different depending on the explored cell. In the case of the concentration-based approach, the background is estimated by the algorithm and, if this background is low, the source strength needs to be known to within one order of magnitude. If the background is high, the algorithm behaves more robustly in the range studied as compared to the binary case except in the first case (the assumed source strength is very small).

### 3.2. Results for the Real Experiments

**Figure 9:**
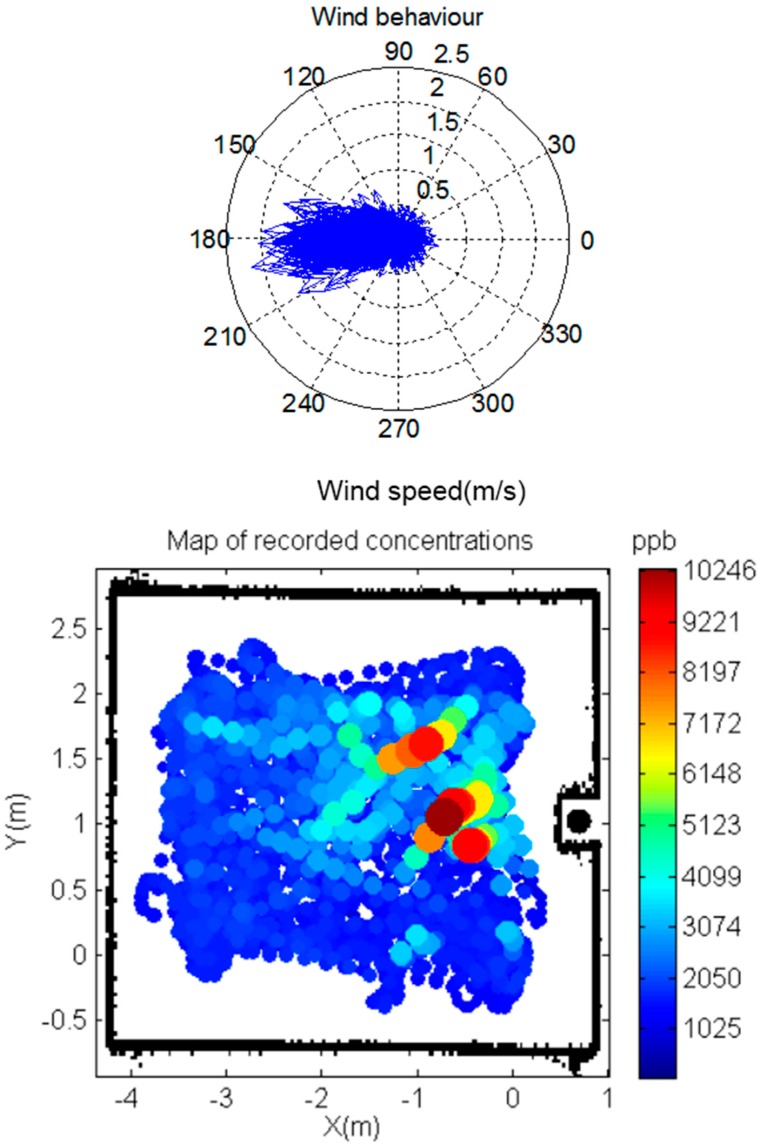
(**Top**) a representative wind distribution recorded within the designed scenario during a random exploration maneuver. Both, the direction (degrees) and the speed (m/s) of the wind are indicated. (**Bottom**) map of concentrations recorded with the PID. Source location at (10, 4) is indicated by a black circle.

First we will give some details concerning the wind distribution within the exploration arena. Figure 9 (top) shows a characterization of the behavior of the wind within the designed scenario for one of the experiments that was carried out. The median wind speed was 0.28 [0.08 − 1.12] m/s. Also, there was a predominant wind direction although with a scatter on the direction angle of approximately 60 degrees. Similar behavior of the wind was observed in all the experiments. The map of concentrations showed in Figure 9 (bottom) is consequence in part of such airflow generated.

**Figure 10:**
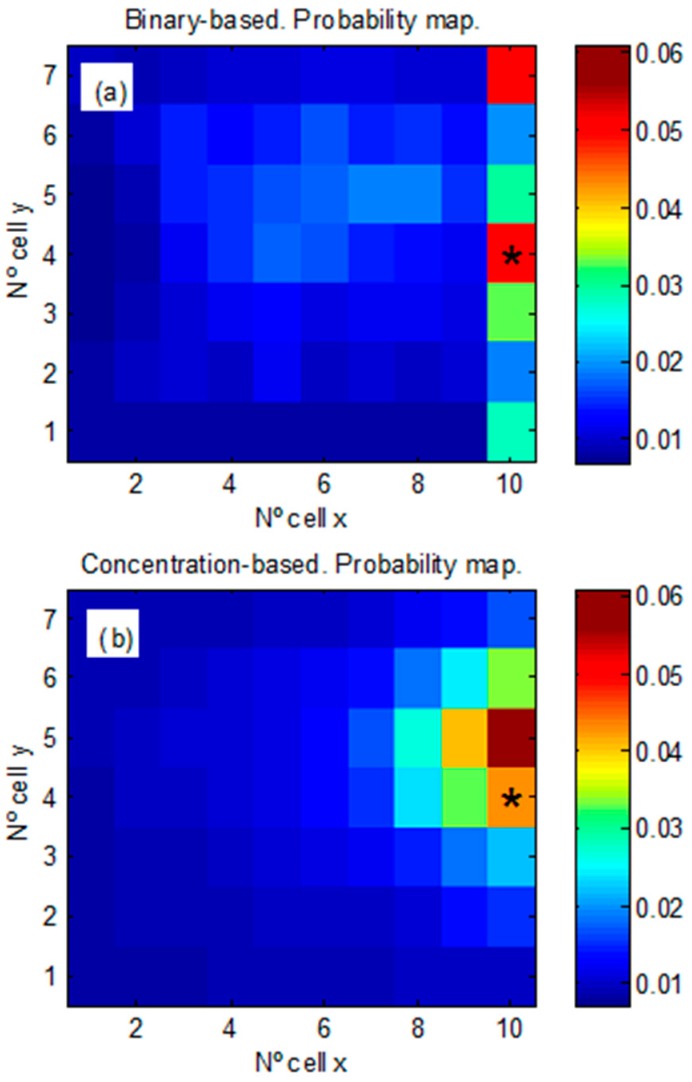
Mean probability maps at the end of the exploration time for the set of real experiments. Source location at (10, 4) is marked with an asterisk. (**a**) Binary-based approach. (**b**) Concentration-based approach.

**Figure 11:**
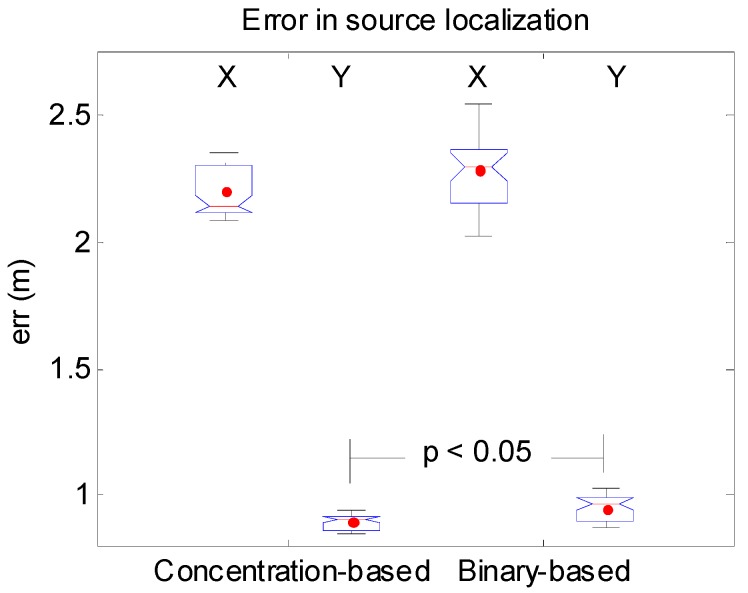
Errors in source localization in both the X and the Y directions for both algorithms. Statistically significant difference is indicated.

The averaged probability maps (over the 10 experiments) are shown in Figure 10. The mean probability assigned by each algorithm to the real source location is practically identical, P = 0.045 [0.030 − 0.076] and P = 0.050 [0.028 − 0.084] for the concentration-based and for the binary-based respectively. However, the probability map for the binary-based algorithm (Figure 10a) exhibits more variance in the localization of the real source position, as it is observed in Figure 10a. For the concentration-based algorithm (Figure 10b), relatively stable higher values of probability around the real source location are obtained. The Wilcoxon rank test is also used to compare the errors obtained for each algorithm. In the X direction, the error for the concentration-based algorithm (2.14 [2.09 − 2.35]) m. is lower than for the binary-based one (2.30 [2.03 − 2.55]) m., but without statistical significance. In the Y direction, the error for the concentration-based (0.91 [0.85 − 0.95]) m. is also lower than for the binary-based (0.97 [0.86 − 1.03]) m. and in this case is statistically significant (see Figure 11). 

Results obtained here are in the same line as those obtained for the simulation experiments but the results are not as clear. In the designed scenario, it is likely that some of the assumptions of our algorithm (see Table A2) are not fully satisfied. The wind was not completely uniform in the room: it was much stronger near the source than at the opposite wall and there were some recirculation at the lateral walls. As a consequence the real distribution of the time-averaged concentration deviated from Gaussianity. We think it might be a reason to explain the slight differences observed when comparing both algorithms, in contrast to the strong differences observed in the performance of both algorithms for the simulation experiments. 

### 3.3. Computatitonal Cost

Finally, we have studied the computational cost of the algorithm. It is true that the computational cost is higher than the binary approach, mainly due to need of computing the convolution between the PDF of the background and the PDF of the plume for every cell. The cost is about three times the cost of the binary approach in our current implementation (without optimization). However, our studies have confirmed that the computational cost of each iteration in the algorithm increases very slowly with the number of cells. Moreover, the time to locate the source increases in a linear way approximately with the number of cells.

## 4. Conclusions

In the present paper, modifications of a previously described (binary-based) algorithm have been introduced. The original algorithm can be easily extended to work with multiple mobile sensors. All the information from the mobile sensors can be integrated in the algorithm, whatever their positions are. The algorithm only needs to know in which cell the concentration readings were obtained, then a probability map will be recursively updated. Moreover, the mobile sensors do not need to solely perform plume tracking and might be used for other tasks. 

Additionally, in a real scenario, pollution and some interfering substances may appear in the background, increasing the number of false alarms. Unlike the binary-based algorithm, which uses a threshold to assess whether a concentration is considered as a detection or non-detection event; our algorithm, based on continuous concentrations, builds a background model to assess whether a concentration comes from the background or from a source located further away. Simulation results show that our algorithm behaves much more robustly in the presence of false alarms and better estimates the real source location.

All concentration readings are considered in our algorithm, incorporating them in a continuous manner instead of just using them as binary detections above a certain threshold. This fact removes the need for a threshold level, thereby reducing the number of false alarms (a background model is estimated) and the number of missed detections thus improving the performance of the algorithm proposed by Pang and Farrell. A sensitivity study regarding robustness of the algorithm against deviations from the true value has been presented. It has been shown, the results improve significantly using the concentration-based algorithm if the source strength can be estimated.

Finally, experiments with real data have shown that the concentration-based algorithm seems to perform slightly better than the binary-based one, confirming our results obtained for the simulation experiments. A critical point in our proposal is that the algorithm assumes that the source strength is known. Thus, estimating the source strength would be a promising direction for future research. Results from real experiments show that when the plume dispersion model hypothesis do not hold, both algorithm are still able to perform the estimation tasks although the differences diminish. 

The described algorithm is available in MATLAB code from the authors under request.

## Supplementary Materials

The supplementary materials are available online at http://www.mdpi.com/1424-8220/17/4/904/s1. Images of the robot, the exploration arena with the fans and the used chemical source are available as supplementary materials.

## Appendix A. Supplementary Tables

**Table A1:** Summary of the notation used for the concentration-based approach.

Nc	Number of Cells in the Grid Area
Lx	Length of each cell along the *x*-axis
Ly	Length of each cell along the *y*-axis
c	Instantaneously measured concentration
cb	Concentration contribution due to the background
cp	Concentration contribution due to the chemical plume
$\bar{c}$	Mean concentration at a fixed location
*q*	Source strength or release rate
*U_a_*	Mean wind speed in the downwind direction
*σ_y_*	Diffusion coefficient in the crosswind direction
*σ_z_*	Diffusion coefficient in the vertical direction
$\hat{c}$	Array of all possible instantaneous concentrations *c*
*γ*	Intermittency factor related to the chemical plume
*M*	Mean concentration of a series of readings at a fixed location
$\sigma_{SD}$	Standard deviation of a series of readings at a fixed location
*N_b_*	Number of readings stored in the concentration buffer per each sensor
*c_j_*	Measured concentration within cell *j*
*A_i_*	Event “there is a chemical source within cell *i*”
*B(t_k_)*	Sequence of measured concentrations along the trajectory of the robots until time *t_k_*
$P(A_{i})$	Prior probability of the presence of a chemical source within cell *i*
$P(c_{j}|A_{i})$	Probability that the measurement within cell *j* is due to the addition of the background at cell *j* and a chemical plume due to a source within cell *i*
$P(c_{j}|{\bar{A}}_{i})$	Probability that the measurement of chemical at cell *j* is not due to a source emitting at cell *i*, thus *c_j_* is due to the current background at cell *j*
$S_{ij}(t_{k})\equiv P(A_{i}|c_{j})$	Probability of having a source in cell *i* given that a certain amount of chemical was measured at cell *j* at time *t_k_*
$\beta_{ij}(t_{k})$	Normalized probability (over all cells) of having a chemical source within cell *i* based on a single measured concentration within cell *j* at time *t_k_*
$\alpha^{'}{}_{ij}(t_{k})$	Normalized probability (over all cells) of having a chemical source within cell *i* based on the sequence of measured concentrations along the trajectory of the robots (index *j*) until time *t_k_*

**Table A2:** Main assumptions of the concentration-based algorithm.

For the Plume
Gaussian distribution for the time-averaged concentration
concentration fluctuations governed by turbulences
continuous release, source strength known *q* = 2.90 g/s
one uniquely source at the position (400 m, 400 m)
height, *z* = 2 m
**For the Background**
background is always present
background relatively constant for the exploration time
mean background level can change from one cell to another
no intermittency
**Other Assumptions**
uniform wind field over the exploration time, speed *U_a_* = 2.5 m/s, and 45° direction
neutral atmospheric stability
no deposition of the substance on surfaces
cells are equally likely to contain the source at time *t_0_*
height of sensors is 2 m
response-time of sensors faster than the typical 10min time-average of GPM
