# Supplementary material for: Chemical Source Localization Fusing Concentration Information in the Presence of Chemical Background Noise
Source: Sensors (Basel). 2017 Apr 20;17(4):904. doi: 10.3390/s17040904 (PMC5426828; doi:10.3390/s17040904)
Supplement: Supplementary file 1 [file sensors-17-00904-s001.pdf]

Supplementary Materials: Chemical Source Localization Fusing Concentration Information in the Presence of Chemical Background Noise

Víctor Pomareda, Rudys Magrans, Juan M. Jiménez-Soto, Dani Martínez, Marcel Tresánchez, Javier Burgués, Jordi Palacín and Santiago Marco

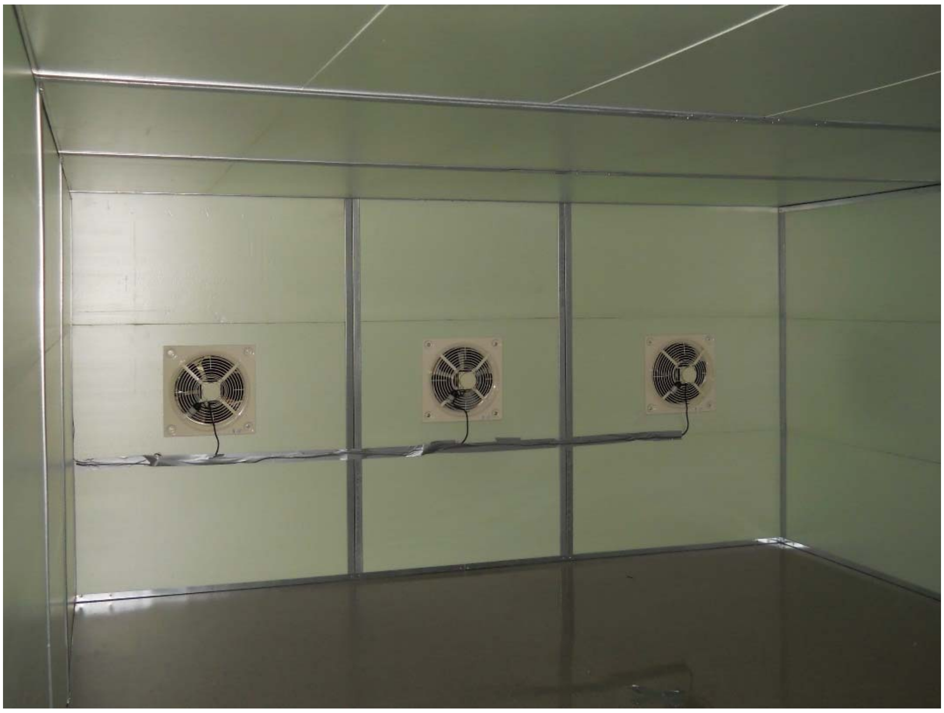

Figure S1. Picture of the exploration arena featuring the three extraction fans.

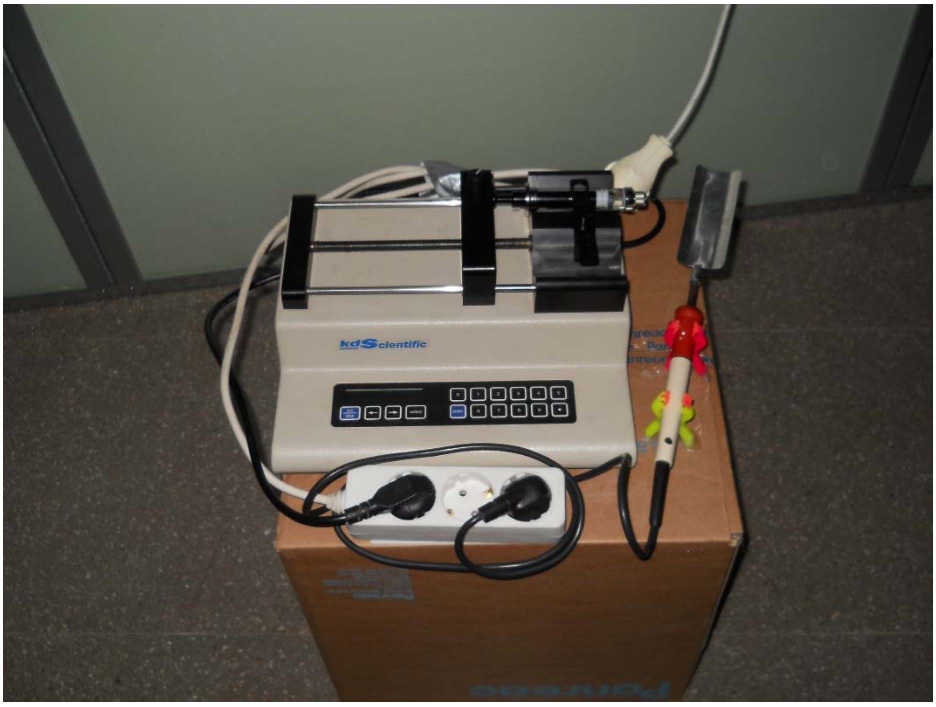

Figure S2. Chemical delivery system based on a KDS-200 injection pump. Drops fall in a hot plate for immediate vaporization.

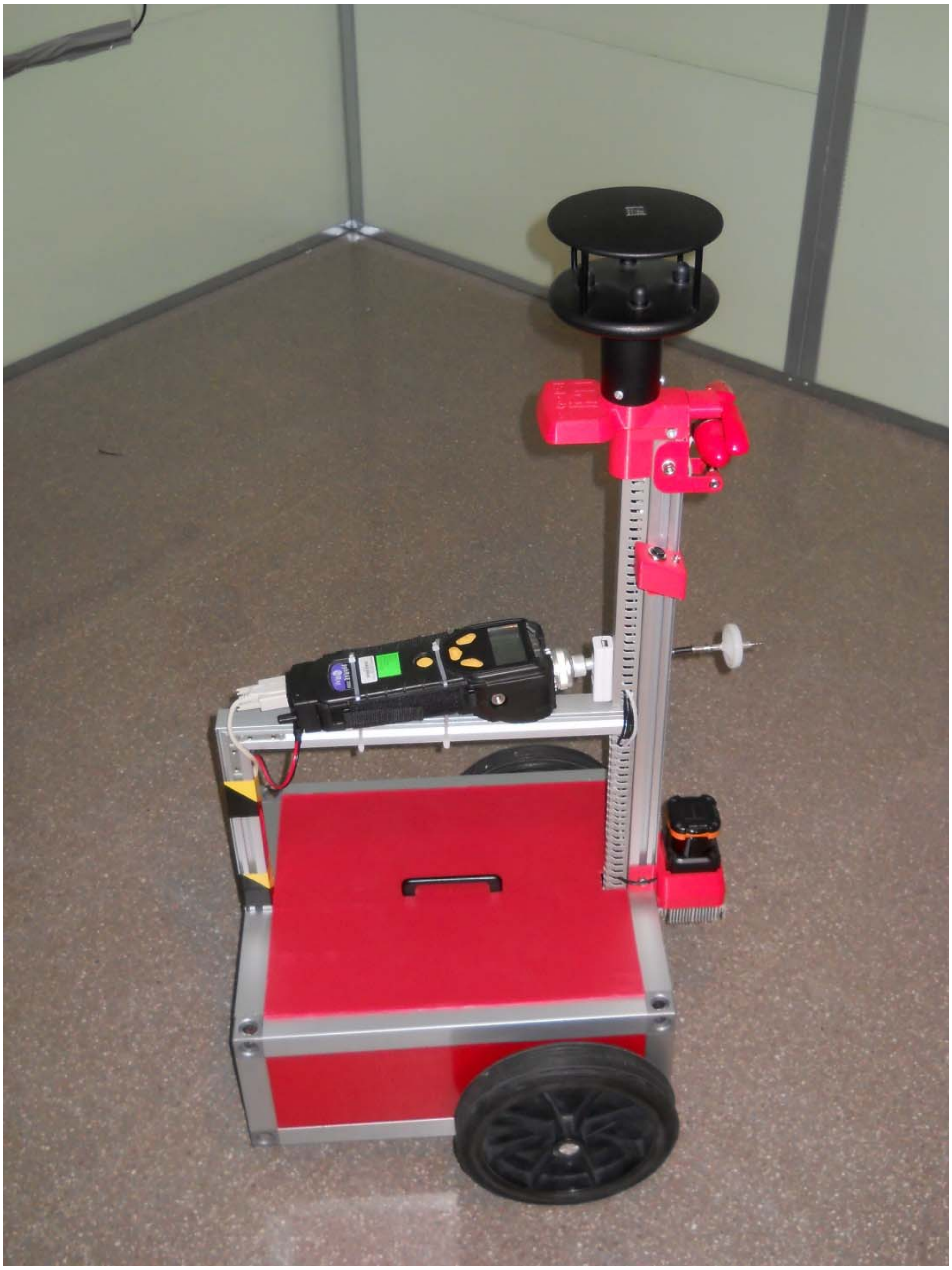

Figure S3. Autonomous robot featuring the wind sensor and the photoionization detector.
